# Antioxidant Potential of Antiviral Drug Umifenovir

**DOI:** 10.3390/molecules25071577

**Published:** 2020-03-30

**Authors:** Elena V. Proskurnina, Dmitry Yu. Izmailov, Madina M. Sozarukova, Tatiana A. Zhuravleva, Irina A. Leneva, Artem A. Poromov

**Affiliations:** 1 Research Centre for Medical Genetics, ul. Moskvorechye 1, Moscow 115522, Russia; 2 Faculty of Fundamental Medicine, Lomonosov Moscow State University, Lomonosovsky prospekt 27-1, Moscow 119234, Russia; dizm@mail.ru (D.Y.I.); videreni@yandex.ru (T.A.Z.); 3 Kurnakov Institute of General and Inorganic Chemistry, Russian Academy of Sciences, Leninsky prospekt 31, Moscow 119991, Russia; s_madinam@bk.ru; 4 Department of Experimental Virology, Mechnikov Research Institute for Vaccines and Sera, Malyi Kazennyi pereulok 5a, Moscow 105064, Russiaaap1309@gmail.com (A.A.P.)

**Keywords:** Umifenovir, antioxidant potential, kinetic chemiluminometry

## Abstract

Free radical reactions play an important role in biological functions of living systems. The balance between oxidants and antioxidants is necessary for the normal homeostasis of cells and organisms. Experimental works demonstrate the role of oxidative stress that is caused by influenza virus as well as the toxic effects of some antiviral drugs. Therefore, antiviral drugs should be characterized by its pro- and antioxidant activity, because it can affect its therapeutic efficiency. The aim of the study was to quantify the antioxidant capacity and propose the mechanism of the antioxidant effect of the antiviral drug Umifenovir (Arbidol^®^). The kinetic chemiluminescence with the 2,2’-azobis (2-amidinopropane) dihydrochloride + luminol system was used to quantify the antioxidant capacity of Umifenovir relative to the standard compound Trolox. With computer simulation, the reaction scheme and rate constants were proposed. The antioxidant capacity of 0.9 μM Umifenovir (maximum concentration of Umifenovir in blood after oral administration of 200 mg) was as high as 1.65 ± 0.18 μM of Trolox. Thus, the total antioxidant capacity of Umifenovir is comparable to the antioxidant capacity of Trolox. Unlike Trolox, Umifenovir reacts with free radicals in two stages. For Trolox, the free radical scavenging rate constant was *k* = 2000 nM^−1^ min.^−1^, for Umifenovir *k*_1_ = 300 nM^−1^min.^−1^, *k*_2_ = 4 nM^−1^min.^−1^. Slower kinetics of Umifenovir provides the prolonged antioxidant effect when compared to Trolox. This phenomenon can make a serious contribution to the compensation of oxidative stress that is caused by a viral disease and the therapeutic effect of the drug.

## 1. Introduction

Free radical reactions play an important role in the biological functions of living systems. The balance between prooxidants and antioxidants is necessary for the normal homeostasis of cells and organisms. Oxidative stress plays an important role in many pathological processes, including viral infections, such as influenza [1]. Excessive reactive oxygen species (ROS) cause oxidative damage of lipid membranes and mitochondrial respiratory chain. Mitochondria act as a platform for antiviral innate immunity. Mitochondrial antiviral signaling involves the activation of the retinoic acid-inducible gene I-like receptors, which requires oxidative phosphorylation activity. The cells with respiratory defects exhibited severely impaired virus-induced induction of interferons and proinflammatory cytokines. Mice with respiratory chain defects were highly susceptible to viral infection and exhibited significant lung inflammation [2]. Oxidative and nitrosative stress may also contribute to reduced antiviral immunity by altering the MDA-5/IRF-3/phosphoIRF-3 axis, as well as contributing to the mechanisms of inflammatory reaction via increased NF-kappaB, and to the augmented turnover rate of thymocyte cells via Bcl2/Bax up-regulation [3]. On the other hand, antiviral drugs may themselves exhibit genotoxic effects as a result of the excessive production of reactive oxygen species (ROS) [4].

Therefore, antiviral drugs with antioxidant potential would protect cells by inhibiting lipid peroxidation and/or preventing the oxidative damage of mitochondrial respiratory chain. The antiviral function of the antioxidant might also involves modulation of multiple signaling pathways/targets useful to viral replication [5]. In fact, many efficient antioxidants exhibit potent antiviral effect [6,7,8,9]. The efficiency of antiviral drugs can be increased in combination with some antioxidants. As an example, the in vitro synergistic antiviral effect of interferon alpha-2b in combination with unithiol on various variants of Herpes simplex was demonstrated [10]. A mixture of echinochrome A, ascorbic acid, and alpha-tocopherol (5:5:1) showed higher antioxidant and antiviral effects than echinochrome A [11]. The intraperitoneal injection of the natural antioxidant silymarin to mice neutralized genotoxic effects of Ribavirin towards mitochondrial DNA [4]. Silymarin also exhibited potent antiviral activity against Mayaro virus and reduced the levels of malondialdehyde and carbonyl protein, which are biomarkers of oxidative stress [12]. 

Influenza often causes pneumonia. Virus damages epithelial cells of the low respiratory tract due to the viral replication in the columnar ciliary epithelium of lungs, which leads to progressive damage of the alveolar cells, bronchopneumonia (viral or combined viral-bacterial), and massive bronchitis. Reactive oxygen species and ROS-producing NADPH oxidase are relevant to virus-induced epithelial apoptosis and lung injury [13]. A combination of antiviral drugs and pyran-SOD conjugates reduced ROS production in alveolar macrophages of influenza-infected mice dramatically and protected the animals from death [14].

To sum up, the oxidative balance is critical in maintaining normal functioning of a host, whereas oxidative stress that is caused by virus affects the intracellular redox balance, which leads to significant changes in the defense system. This provides a therapeutic option for the prevention and control of virus infection. Antiviral drugs should be characterized by its pro- and antioxidant activity, because it can affect the therapeutic efficiency.

Umifenovir (C_22_H_25_BrN_2_O_3_S, ethyl 6-bromo-4-[(dimethylamino)methyl]-5-hydroxy-1-methyl-2- [(phenylsulfanyl) methyl]-1*H*-indole-3-carboxylate) is an oral antiviral drug that is licensed for the treatment and prophylaxis of influenza A and B virus infections in Russia in 1993 (Arbidol^®^, OJSC «Pharmstandard-Leksredstva») and in China in 2006. Umifenovir is rapidly absorbed and distributed to organs and tissues. The maximum concentration in blood plasma after the administration of a single dose of 200 mg is about 415 ng/mL (approximately 0.9 μM) [15]. Umifenovir interacts with the hemagglutinin (HA) protein of influenza viruses, stabilizes it against the low pH transition to its fusogenic state, and it inhibits HA-mediated membrane fusion during influenza virus infection [16].

The antiviral activity of Umifenovir has been shown in vitro and in vivo for different viruses, including influenza types A and B, as well as other acute respiratory tract infection agents (adenovirus, respiratory syncytial virus, coronavirus or SARS virus, rhinovirus, parainfluenza virus) [17,18,19]. Umifenovir inhibits various influenza A virus strains, including Rimantadine- and Ozeltamivir-resistant variants, as well as influenza B viruses (IC50 2–8.5 μg/mL), including pandemic influenza A/California/04/2009(H1N1), A/California/07/2009(H1N1), and A/Moscow/01/2009(H1N1)swl viruses in the cultured MDCK cells (IC50 = 1.5–4.0 μg/mL), highly pathogenic avian A(H5N1) viruses with EC50 ranging from 7.2 to 23.0 µM. The lungs of the mice that were treated with Umifenovir had less severe histopathologic lesions when compared to the control group [20]. Umifenovir possesses micromolar-level anti-viral effects (EC50 values that range from 10.57 +/− 0.74 to 19.16 +/− 0.29 µM) in Vero cells infected with Zika virus, West Nile virus, and tick-borne encephalitis virus. Therefore, Arbidol could be a promising therapeutic agent in the selective treatment of flaviviral infections [21]. The sensitivity to Umifenovir of influenza viruses circulating in the 2012–2014 seasons, as well as the sensitivity of influenza A virus isolated from patients, has been proven in the ARBITR study [18]. All 18 clinical isolates of influenza A viruses that were obtained before and during therapy were susceptible to Umifenovir with 50% effective concentration ranging from 8.4 +/− 1.1 to 17.4 +/− 5.4 μM without the development of drug-resistant variants [22]. Experimental studies at concentrations of 25–100 mg/mL proved the direct antiviral activity of Umifenovir on the early viral replication of severe acute respiratory syndrome (SARS) virus in the cultured GMK-AH-1 cells [23]. 

Umifenovir (Arbidol) is licensed and widely used in Russia for the prophylaxis and/or treatment of influenza. The clinical trials of Umifenovir were performed in the former USSR during 1980–1995 and post-marketing phase IV trial in 2011–2017 influenza seasons. In a double-blind, randomized, placebo-controlled clinical study ARBITR (ClinicalTrials.gov Identifier: NCT01651663) investigating the efficacy and safety of Arbidol among 359 adults that was carried out in Russia, Umifenovir (800 mg daily for five days) significantly (*p* < 0.05) reduced the duration of fever (68 h in Umifenovir group and 75.3 in placebo group), muscle pain (52.2 vs 59.1), and weakness (76.9 vs 88.9) in Umifenovir-treated group when compared to control untreated patients, and reduced the risk of complications, namely influenza with low respiratory tract infections (0% vs 3.36%) [24]. Post-marketing surveillance of the efficiently of Umifenovir in clinical use was made by retrospective analyzed of 5287 patients with influenza and other ARVI in 88 hospitals from 50 regions of the Russian Federation. In patients that were treated with Umifenovir (in the first 48 h after disease onset), the duration of fever and frequency of pneumonia proved to be lower than those in the patients who did not receive antiviral therapy. Umifenovir therapy substantially reduces the duration of fever and risk of complications, especially in patients with laboratory-confirmed influenza infection [25]. 

The prophylactic effect of Arbidol was also studied in a randomized placebo-controlled trial in children. The study was conducted in 1995 by Pasteur Institute in St. Petersburg and it included 155 children who received Arbidol twice a week for three weeks before the peak of influenza morbidity. Arbidol prophylaxis 1.2-4-fold reduced the overall morbidity (depending on the age group), and the duration of illness was also decreased by 1.8–3.5 days [26]. 

Arbidol effectiveness for preventing and treating influenza might be due to the co-existence of its two actions: (1) specific antiviral activity against influenza and other respiratory viruses; (2) interferon-inducing and immune-modulating properties, which was shown on cultured cells, animals [27], and humans [28]. Arbidol-treated patients with lower baseline immunity showed improvement in immunological parameters (number of CD4, CD8 lymphocytes and B lymphocytes, and concentration of serum immunoglobulins). The administration of a single oral dose of Umifenovir 0.1 g by healthy adult volunteers resulted in the induction of serum interferon up to 40–80 interferon units/mL. For children, the administration of 0.01 g/kg/day leads to the 5.3-fold increase in the endogenous interferon production in 70% of subjects [28]. There is some evidence that Umifenovir helps the phagocytic action of macrophages [29]. The presence of not only direct antiviral action, but also other indirect effects was the reason for the investigation of antioxidant activity of Umifenovir.

**Figure 1:**
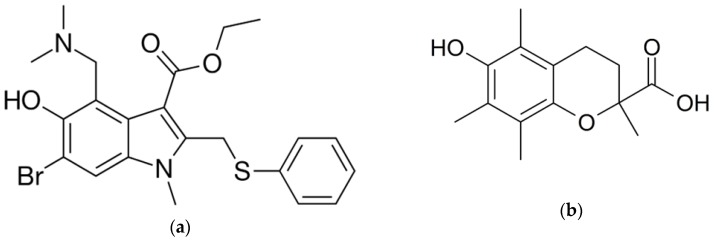
The structures of (**a**) Umifenovir and (**b**) Trolox.

It is a free hydroxyl group that provides the antioxidant activity of Umifenovir (Figure 1). Previously, the antioxidant effect of Umifenovir to prevent lipid peroxidation was studied in vitro [30]. The authors proved this substance to be a lipid antioxidant. However, lipid peroxidation does not restrict oxidative stress. The assessment of antioxidant potential in relation to ROS-reactions in the aqueous phase is no less important, because non-lipid ROS may cause oxidative modification of proteins and DNA. More studies are needed to characterize comprehensively the antioxidant properties of Umifenovir.

The conventional approach is based on the quantitative comparison with a reference compound to assess the antioxidant capacity of a substance. This parameter reflects the ability of the substance to scavenge free radicals. For example, the TRAP method (Total Radical-Trapping Antioxidant Potential) is widely used, which is based on scavenging free radicals that formed by thermolabile azo-compounds [31]. However, this method does not take the physicochemical characteristics of the antioxidant into account. Another strategy is based on the evaluation of the rate constants of the interaction of the antioxidant with the radicals [32,33].

In this work, we studied the antioxidant properties of Umifenovir with the modified TRAP protocol [34]. While using computer simulation of chemical kinetics, the scheme of antioxidant reactions and the rate constants were proposed.

## 2. Results

### 2.1. The Antioxidant Capacity of Umifenovir Assessed by Modified TRAP Method

**Figure 2:**
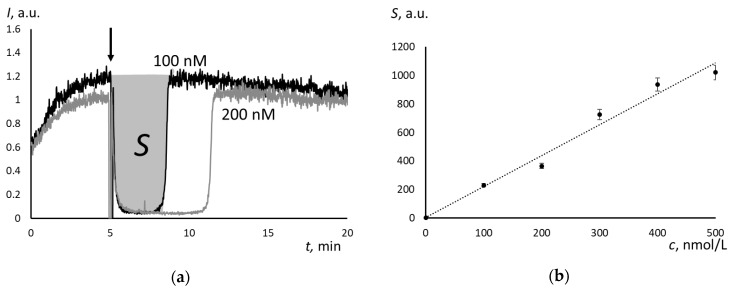
(**a**) Chemiluminograms for Trolox (concentrations are indicated in the figure), the chemiluminescent system consisted of 2.5 mM ABAP and 2 μM luminol, the arrow shows the moment of the addition of Trolox, shaded is the area of the depression that is proportional to the number of scavenged free radicals; and, (**b**) The calibration plot of the depressed area on Trolox concentration, *n* = 5.

The steady-state production of free radicals was provided by the decomposition of 2,2′-azobis (2-amidinopropane) dihydrochloride (ABAP) at 37 °C. Luminescence decreases to the background level almost instantly when Trolox was added to the ABAP + luminol mixture. After the certain interval (a latent period), Trolox was completely exhausted, and luminescence returns sharply until the initial level (Figure 2a). The area of the luminescence depression reflects the total amount of scavenged radicals. It linearly depended on Trolox concentration (Figure 2b). The calibration equation is:

*S* = (2.16 ± 0.04)*c* + (3.82 ± 0.02), *r* = 0.988 (*P* = 0,95; *n* = 6) (1)

where *c* is Trolox concentration, nM.

**Figure 3:**
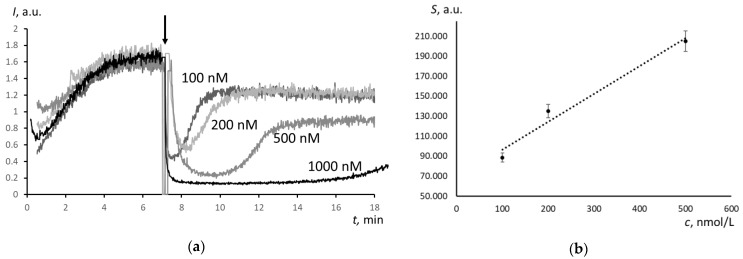
(**a**) Chemiluminescence plots for Umifenovir (concentrations are indicated in the figure), the chemiluminescent system consisted of 2.5 mM ABAP and 2 μM luminol, the arrow shows the moment of the addition of Umifenovir; (**b**) The calibration plot of the area of the “fast” depression on the Umifenovir concentration, *n* = 5.

For Umifenovir, the chemiluminograms were of another type. The depression was incomplete, and after depression, the stationary level was lower that the initial one (Figure 3a). Therefore, the mechanisms of free-radical scavenging of Trolox and Umifenovir are different. According to these curves, the scavenging effect of Umifenovir involves at least two phases: “fast” and “slow”.

The area of depression *S* (“fast” part only) linearly depends on the Umifenovir concentration (Figure 3b):

*S* = (0.27 ± 0.08)*c* + (68.8 ± 17.3), *r* = 0.986 (*P* = 0,95; *n* = 5) (2)

where *c* is Umifenovir concentration, nM.

**Figure 4:**
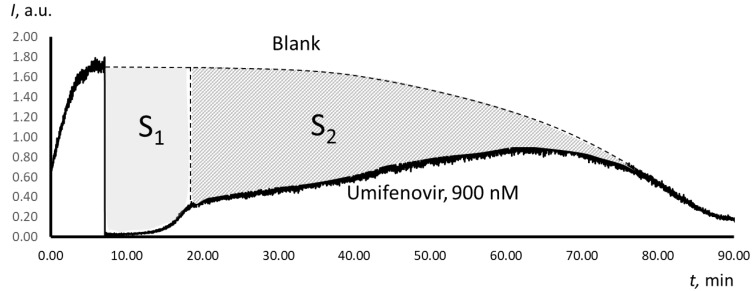
Calculation of the total antioxidant potential *S* of Umifenovir as a sum of *S*_1_ and *S*_2_, where *S*_1_ is the area of the “fast” part of chemiluminescence depression, *S*_2_ is the area of the “slow” part of chemiluminescence depression. The plots are obtained for 900 nM that is a maximum concentration of Umifenovir in blood after oral administration of 200 mg.

The Trolox-equivalent antioxidant capacity of a substance can be determined using TRAP or TAR (Total Antioxidant Reactivity) methodology [30]. The TAR index is obtained from the instantaneous decrease in luminescence after adding the antioxidant, while the TRAP index is calculated from the latent period. However, for Umifenovir, both methods are not applicable, as they do not take the “slow” part of the scavenging effect into account. We propose to use the area of total depression *S* as a measure of the antioxidant capacity of Umifenovir (Figure 4). The area of depression is defined as an integral of the difference between the blank and analytical plots. It is a sum of the area of “fast” depression *S*_1_ and “slow” depression *S*_2_. The areas were calculated while using the specially designed features of PowerGraph 3.3 software.

Table 1 provides the calculated antioxidant capacity values for Umifenovir in μM of Trolox.

### 2.2. The Antioxidant Activity of Umifenovir Studied with Computer Simulation

Computer simulation was used to estimate the rate constants of antioxidant reaction of Umifenovir (the antioxidant activity). For the supposed reactions and given initial concentrations of the reactants, the rate constants were varied to achieve maximum fitting of the calculated and experimental plots. Figure 2a and Figure 3a show the experimental curves that were obtained for 100 nM Trolox and Umifenovir used for the computer simulation, respectively.

To simulate a blank stationary level of chemiluminescence, a simple model that consists of two reactions was proposed: (1) the reaction of free radical generation and (2) the chemiluminescence reaction:

(1) ABAP + Lum → R• (rate constant *k*_R_)
(2) R• → P + light, (*k*_Lum_)

where Lum is a luminol molecule, R• is a free radical or a reaction product in the excited state with which the antioxidant reacts, P is a stable product of the free-radical reaction (here and below).

To simulate the effect of antioxidants, a reaction for Trolox was added to the set:

(3) R• + In → P (*k*_In_),

where In is an antioxidant (inhibitor).

Two reactions should be added for Umifenovir:

(3’) R• + In_1_ → P (*k*_In1_)
(4) R• + In_2_ → P (*k*_In2_)

**Figure 5:**
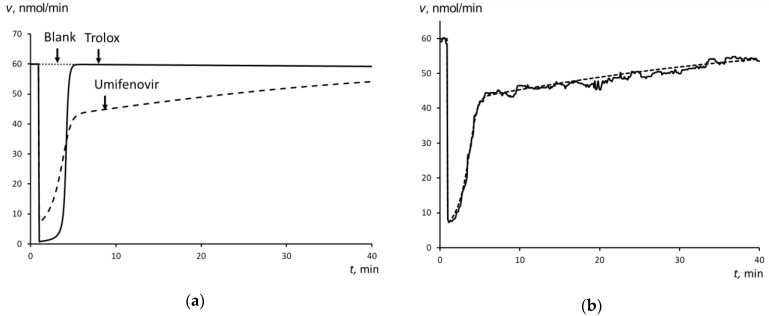
(**a**) The simulated chemiluminescence plots, spot line—ABAP without antioxidant (blank), dotted line—the chemiluminogram of Umifenovir, solid line—the chemiluminogram of Trolox; (**b**) The experimental (solid line) and simulated (dashed line) plots for Umifenovir.

Figure 5a shows the calculated data for Trolox and Umifenovir, Figure 5b shows the combined calculated and the experimental plots for Umifenovir.

For Trolox, the rate constant of free radical scavenging were estimated: *k*_In_ = 2000 nM^−1^min^−1^. For Umifenovir, the rate constants were estimated: *k*_In1_ = 300 nM^−1^min^−1^, *k*_In2_ = 4 nM^−1^min^−1^.

## 3. Discussion

It was previously shown that the effect of any antioxidant on the chemiluminescence kinetics can be described by a single reaction between the antioxidant and a free radical [35]:

R• + In→ P  (*k*_In_)

**Figure 6:**
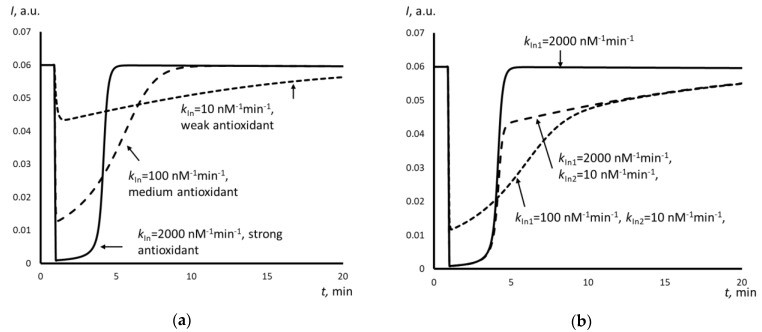
(**a**) The simulated chemiluminescence plots for the one-stage mechanism: solid line corresponds to a strong antioxidant, *k*_In_ = 2000 nM^−1^min^−1^, dotted line—a medium antioxidant, *k*_In_ = 100 nM^−1^min.^−1^, dashed line—a weak antioxidant, *k*_In_ = 10 nM^−1^min.^−1^; (**b**) the simulated chemiluminescence plots for the two-stage mechanism: solid line corresponds to a strong antioxidant, *k*_In1_ = 2000 nM^−1^min^−1^, dotted line—a strong antioxidant, *k*_In1_ = 2000 nM^−1^min.^−1^, *k*_In2_ = 10 nM^−1^min.^−1^, dashed line—a medium antioxidant, *k*_In1_ = 100 nM^−1^min.^−1^, *k*_In2_ = 10 nM^−1^min.^−1^.

In this case, the chemiluminescence kinetics strongly depends on the rate constant *k*_In_. Depending on the *k*_In_ value and the chemiluminescence system used, all of the antioxidants can be classified as strong, medium, and weak (Figure 6a). Strong antioxidants are characterized by high rate constants and quench the chemiluminescence sharply and completely for a fixed time interval, which is referred to a “latent period”. The complete exhaustion of the antioxidant following with a sharp increase in the luminescence causes the end of the latent period, which returns the previous stationary level. As an example, Fig. 6a presents a simulated chemuliminogram for an antioxidant with *k*_In_ = 2000 nM^−1^min.^−1^. Weak antioxidants with low rate constants (for example, *k*_In_ = 10 nM^−1^min^−1^ in Figure 6a) do not give the latent period. Instead, the luminescence decreases slightly. The effect of weak antioxidants is prolonged, as they react very slowly. The effect of medium antioxidants (*k*_In_ = 100 nM^−1^min.^−1^ in Figure 6a) is between the effects of strong and weak antioxidants. 

The addition of Trolox to the chemiluminescence system results in the complete depression of chemiluminescence, which is typical for strong antioxidants (Figure 5a). A one-stage scheme with *k*_In_ = 2000 nM^−1^min.^−1^ appeared to be adequate for the successful computer simulation. Trolox can be classified as a strong antioxidant from the kinetics point of view.

For Umifenovir, the chemiluminescence kinetics is more complicated (Figure 3a). First, immediately after the addition of the antioxidant, a significant but incomplete decrease in intensity occurs, which is typical for antioxidants of medium strength. Second, there is a stage of a slight decrease of the intensity of chemiluminescence with a slow and long rise of the luminescence to a stationary level, which is typical for weak antioxidants. Thus, a two-stage model is needed, which consists of two consequent antioxidant reactions with different rate constants. Figure 6b gives the examples of simulated plots for abstract antioxidants. The computer simulation confirmed the two-stage mechanism of the antioxidant effect of Umifenovir. The found rate constants 300 nM^−1^min.^−1^ and 4 nM^−1^min.^−1^ are characteristic for medium and weak antioxidant. Hence, from the kinetics point of view, Umifenovir can be classified as a medium antioxidant. The difference between the mechanisms of action for Trolox and Umifenovir makes it impossible to TRAP and TAR methodologies for the quantification of antioxidant capacity of Umifenovir. As a measure of antioxidant capacity, we propose using the total area of depression of the chemiluminescence, including “fast” and “slow” parts of the chemiluminograms. For 0.9 μM of Umifenovir, which corresponds to the maximal concentration in blood after oral administration of 200 mg, the Trolox-equivalent antioxidant capacity was as high as 1.65 μM. Hence, from the thermodynamic point of view, Umifenovir is a more effective antioxidant than Trolox in 1.65/0.9 = 1.8 times. Note that the effect of Umifenovir lasts for about an hour (Figure 4), while the effect of 0.9 μM Trolox lasts for about 12 minutes. Thus, Umifenovir acts five-fold longer than Trolox.

Let us compare our results to the data that were obtained by Vasil’eva at al. [30]. Using a chemiluminescence system consisting of phospholipid liposomes, FeSO_4_, and coumarin C 525 in Tris buffer solution, they determined the antioxidant effect of Umifenovir on lipid peroxidation as a concentration of 50% depression (C50%) of amplitude of slow flash. This value (5 μM) was two orders of magnitude lower than C50% of α-tocopherol (0.06 μM). The authors attribute this fact to a poor solubility of Umifenovir in lipids. Note that, because of the comprehensive mechanism of lipid peroxidation, the amplitude of slow flash correlates with the antioxidant capacity in a complex way and it can only be used for semi-quantitative assessment. Measurements of the amplitude of the fast flash would be more adequate, but it was not possible with the chemiluminometer used by the authors. Based on the latent time of the slow flash, the authors proposed that the antioxidant effect of Umifenovir was due to the reaction with free radicals, but not with iron. However, these conclusions require confirmation by computer simulation, which may be the aim of the next study. In this study, we used 2,2’-azobis(2-amidinopropane)hydrochloride, a well-known water soluble radical initiator, which has been successfully applied as an initiator of lipid peroxidation [36]. This substance forms the aliphatic carbon-centered radical, which is transformed into more reactive peroxyl radical in the presence of oxygen. It is considered to be a good model substance for ozone and UV-B exposure [37]. ABAP, as a radical generator, offers a useful tool for studying oxidative stress in biochemical and biological models and antioxidant properties of substances due to a simple chemical mechanism of the degradation and possibility to work at the physiological temperature. However, such studies can only be performed in aqueous phase, not in lipids.

Summing up, Umifenovir has potent antioxidant effect in relation to organic radicals in the aqueous phase due to two-stage mechanism. This phenomenon can make a serious contribution to the compensation of oxidative stress that is caused by a viral disease and to the therapeutic effect of the drug. Further studies on the therapeutic effect of Umifenovir on animal influenza models in comparison with its antioxidant potential will be of special interest. 

## 4. Materials and Methods

The enhanced chemiluminescence protocol quantified the antioxidant activity of Umifenovir. The chemiluminescent system consisted of a source of free radicals 2,2’-azobis (2-amidinopropane) dihydrochloride (ABAP, Sigma, Saint Louis, MO, USA) and a chemiluminescent probe luminol (Sigma). The method was described elsewhere [34]. A luminol solution of 1 mM (Sigma, Saint Louis, MO, USA) and ABAP solution of 50 mmol/L was prepared by dissolving the weighed samples in phosphate buffer solution (100 mM KH_2_PO_4_, pH 7.4, Sigma, Saint Louis, MO, USA). The total volume in a cuvette was 1.000 mL. A mixture of ABAP and luminol (final concentrations were 2.5 mM and 2 μM, respectively) was added to a buffer solution (pH 7.4) at 37 °C. The chemiluminescence was recorded until a stationary level had been achieved, and then an aliquot of the antioxidant solution of Trolox or Umifenovir was added. The registration was performed until the new steady-state level was achieved (Figure 2a).

As a reference compound, Trolox (Sigma, Saint Louis, MO, USA), a water-soluble analogue of vitamin E, was used. A stock solution of 100 mM Trolox was prepared in phosphate buffer solution. Working solutions were prepared by the dilution of the stock solution with the buffer solution. Umifenovir (Erregierre S.P.A., San Paolo d’Argon, BG, Italy, M = 477.4 g/mol) was dissolved in acetone (99.9% PanReac, Castellar del Vallès, Barcelona, Spain). 

The measurements were carried out with a 12-channel Lum-1200 chemiluminometer (DISoft, Moscow, Russia). The chemiluminometer provides the detection of visible light within a range from 300 to 700 nm. No light filters were used in our experiments.

Signal processing were performed with PowerGraph 3.3 Professional software (DISoft, Moscow, Russia). The statistical processing of the data was performed with STATISTICA v.10.0 software (StatSoft Inc., Tulsa, OK, USA).

In contrast to TRAP and TAR methodologies, in determining the total antioxidant potential of Umifenovir, we calculated the total area of depression *S* as a sum of *S*_1_ and *S*_2_, where *S*_1_ is the area of depression of the “fast” phase of scavenging free radicals, S_2_ is the area of depression of the “slow” phase of scavenging free radicals (the shaded area in Figure 4). The areas of depression were calculated with PowerGraph 3.3. Professional software. 

The computer simulation was carried out with the specially designed computer program “Kinetic Analyzer” (D.Yu. Izmailov). For a set of the predetermined reactions and the initial concentrations of the reactants, the rate constants were selected providing the maximal fitting of experimental and calculated plots. As a criterion for maximal fitting, the minimum sum of squared residuals was calculated with the OriginPro software (OriginLab, Northampton, MA, USA). 

## Figures and Tables

**Table 1:** The antioxidant capacity of Umifenovir relative to Trolox.

Concentration of Umifenovir	Antioxidant Capacity in μM of Trolox, Mean ± Standard Deviation, *n* = 9
0.1 μM	S_1_ = 0.049 ± 0.004 (“Fast” capacity) S_2_ = 0.095 ± 0.010 (“Slow” capacity) *S* = 0.14 ± 0.12 (Total capacity)
0.9 μM (maximal concentration of Umifenovir in blood after taking 200 mg per os)	*S*_1_ = 0.45 ± 0.04 (“Fast” capacity) *S*_2_ = 1.20 ± 0.13 (“Slow” capacity) *S* = 1.65 ± 0.18 (Total capacity)
